# Recent progress in fiber-based soft electronics enabled by liquid metal

**DOI:** 10.3389/fbioe.2023.1178995

**Published:** 2023-04-28

**Authors:** Bowen Yang, Zihan Yang, Lixue Tang

**Affiliations:** ^1^ Beijing Key Laboratory of Fundamental Research on Biomechanics in Clinical Application, School of Biomedical Engineering, Capital Medical University, Beijing, China; ^2^ Fashion Accessory Art and Engineering College, Beijing Institute of Fashion Technology, Beijing, China; ^3^ Beijing Advanced Innovation Center for Big Data-Based Precision Medicine, Capital Medical University, Beijing, China

**Keywords:** liquid metal, fiber, soft electronics, stretchable conductors, spinning

## Abstract

Soft electronics can seamlessly integrate with the human skin which will greatly improve the quality of life in the fields of healthcare monitoring, disease treatment, virtual reality, and human-machine interfaces. Currently, the stretchability of most soft electronics is achieved by incorporating stretchable conductors with elastic substrates. Among stretchable conductors, liquid metals stand out for their metal-grade conductivity, liquid-grade deformability, and relatively low cost. However, the elastic substrates usually composed of silicone rubber, polyurethane, and hydrogels have poor air permeability, and long-term exposure can cause skin redness and irritation. The substrates composed of fibers usually have excellent air permeability due to their high porosity, making them ideal substrates for soft electronics in long-term applications. Fibers can be woven directly into various shapes, or formed into various shapes on the mold by spinning techniques such as electrospinning. Here, we provide an overview of fiber-based soft electronics enabled by liquid metals. An introduction to the spinning technology is provided. Typical applications and patterning strategies of liquid metal are presented. We review the latest progress in the design and fabrication of representative liquid metal fibers and their application in soft electronics such as conductors, sensors, and energy harvesting. Finally, we discuss the challenges of fiber-based soft electronics and provide an outlook on future prospects.

## 1 Introduction

In recent years, we have seen the rapid integration of electronics, soft textiles, and human tissues. The fusion of electronics with textiles or human tissues requires that electronics be flexible, stretchable, and compatible with human tissues. Soft electronics are usually realized by connecting electronic components using stretchable conductors on soft substrates. Those reported stretchable conductors include intrinsically stretchable conductors and structure-enabled stretchable conductors. The intrinsically stretchable conductors contain liquid metals (KIM et al., 2015; LI et al., 2015; ZHENG et al., 2019), metal-nanomaterials (CHEN and LIAO, 2014; KIM et al., 2014; JUNG et al., 2022), carbon-nanomaterials (DUAN et al., 2016; SCHUTT et al., 2017; WU et al., 2022), and conductive hydrogels (YANG and YUAN, 2019; HU et al., 2023). Stretchable conductors can also be achieved by designing conductors such as gold and copper into curved structure (JAHANSHAHI et al., 2012; WON et al., 2014), serpentine structure (LIN et al., 2017a; JANG et al., 2018) and 3D structure (GAO et al., 2014; JANG et al., 2017). Those conductors mainly serve as interconnects in soft electronics, but also as sensors, heaters, electrodes and antennas (ZHANG et al., 2018; KIM et al., 2022; FAZI et al., 2023). Liquid metals have received extensive attention in the field of soft electronics due to their excellent electrical conductivity, stretchability, and low cost (CAO et al., 2023; KIM et al., 2023). Thus, liquid metals are one of the most economical and commercially promising materials for realizing soft electronics. Another challenge of soft electronics is that the reported soft electronics are usually poor in air permeability. Because, most soft electronics are manufactured with air-impermeable substrate such as, silicones, styrene resins and polyurethanes (HE et al., 2020; PAUL et al., 2022; SHI et al., 2022). In addition, the air permeability of soft electronics will be compromised after encapsulation (KWON et al., 2019; YANG et al., 2022a). To make the electronics soft, air-permeable, and biocompatible, fibers with unique structures and functions have received extensive attention. Substrates composed of fibers are gradually being used in various fields, such as soft electronics, tissue engineering, wearable electronics, and human-machine interfaces. Here we present an overview of recent studies on the applications of fibers in soft electronics for the following reasons. Firstly, substrates composed of fibers have excellent air permeability due to their high porosity (HOMAEIGOHAR and ELBAHRI, 2014), making them ideal materials for the next-generation of soft devices. Secondly, fibers can be woven directly into various shapes, or formed into various shapes on the mold by spinning techniques such as electrospinning, melt spinning and air-jet spinning. In addition, fibers can be coated and modified with functional materials such as liquid metal (WANG et al., 2021), graphene (XU et al., 2013) and metal nanomaterials (VELGOSOVA et al., 2023). By adjusting the parameters of the spinning equipment, the fibers with various structures can be easily manufactured, such as core-shell structures, layered structures, and hollow structures (CHENG et al., 2017a). Thirdly, Spinning can change the mechanical properties of some materials. For example, Oxide ceramics are usually hard and brittle, which will break when bent. By contrast, the TiO nanofibers from TiO-containing spinning sol are bendable and stretchable (ZHANG et al., 2021). Finally, materials from nanofibers usually have excellent biocompatibility. Because substrates from nanofibers can achieve anisotropy and layered structure similar to human tissues, which is suitable for cell growth to reconstruct human tissue, which cannot be reproduced by traditional materials (LI et al., 2022a). This characteristic of nanofibers further promotes the fusion of electronic devices and human tissues. In this paper, the development of spinning technology and its applications in various fields are introduced, the typical application of liquid metals and their patterning strategies are briefly discussed. The fabrication of liquid metal fibers and their application in soft electronics are reviewed. Finally, an outlook on future prospects is also provided.

## 2 Liquid metal enabled soft electronics

### 2.1 Typical applications of liquid metal in soft electronics

In recent years, liquid metal has attracted much attention due to its good electrical conductivity, thermal conductivity, flexibility, low toxicity, and deformability. It is believed that liquid metal shows broad application prospects in 3D printing (DATTA et al., 2020), wearable devices (ZHANG et al., 2020a), soft robots (HOU et al., 2018), *etc.*

As the most famous liquid metal, mercury has a melting point of −38.83°C, and it has been applied in medical and electronic fields such as mercury batteries, mercury lamps, and sphygmomanometers. However, mercury is also known for its toxicity. The mercury vapor at room temperature can be absorbed by the alveoli through respiration. Moreover, it can pass through the blood-brain barrier and affect the human nervous system (CHEN et al., 2022). Thus, the use of mercury in biomedical applications and wearable devices is limited, where the biosafety has been a focus of attention. Some alkali metals such as caesium (Cs, melting point: 28.5°C), rubidium (Rb, melting point: 39°C) and francium (Fr, melting point: 27°C) have melting points just above room temperature, and they are usually used in liquid form. However, such alkali metals are highly reactive and pyrophoric. They react explosively with water even at low temperatures, making them difficult to use in soft electronics (PARK et al., 2021). As an alternative to mercury and highly reactive alkali metals, gallium and gallium-based alloys are more stable, biocompatible, and do not generate vapor at room temperature (COCHRAN and FOSTER, 1962; DICKEY, 2017). Although pure gallium is not liquid (melting point of 29.8°C) at room temperature, metals such as indium, tin, and zinc can be doped into gallium to form gallium alloys to greatly reduce the melting point of gallium. For example, galinstan (68% gallium, 22% indium, and 10% tin by weight) has a melting point of −19°C, GaInZn (72% gallium, 12% indium, and 16% zinc by weight) has a melting point of 17°C, and EGaIn (75.5% gallium and 24.5% indium by weight) has a melting point of 15.5°C (MAJIDI et al., 2017). Those gallium alloys have similar physical properties (a liquid state at room temperature, viscosity ∼2 × 10^−3^ kg/m/s, density ∼6 g/cm^3^, electrical conductivity ∼3 × 10^6^ S/m) (TANG et al., 2022a), when used in soft electronics, most gallium alloys can be substituted for each other.

Ga and Ga alloys are generally considered biocompatible materials (MA et al., 2021; PARK et al., 2021; CHEN et al., 2023) and have many applications in drug delivery (LU et al., 2015a), skin electronics (TANG et al., 2022b), implantable devices (DING et al., 2020), *etc.* The vapor pressure of Ga is close to zero, which ensures that Ga will not enter the human body through breathing (TANG et al., 2021). The Ga are regarded to be non-toxic to mammal cells (LI et al., 2018a; KIM et al., 2018; WANG et al., 2018). The toxicity of the gallium-based alloys is believed from the released Ga ions (MOSCHèNSCHWEIZER et al., 2001). Ga not only reacts with acidic and alkaline solutions, but also slowly reacts with water to produce Ga ions. Researchers evaluated the toxicity of Ga ions and In ions using L929 mouse fibroblasts. The results showed that Ga and In ions did not inhibit mitochondrial dehydrogenase activity, indicating that Ga and In ions did not exhibit significant toxicity (CHANDLER et al., 1994). Research has shown that Ga ions can disrupt the Fe homeostasis in immune cells, regulate the production of NO and pro-inflammatory cytokines by activated immune cells, and have anti-inflammatory effects (SALES et al., 2021; ZHANG et al., 2022a). Gallium nanodroplets upregulate eIF2α Phosphorylation level and inhibit NO synthesis without interfering with Fe homeostasis (ZHANG et al., 2022b). The toxicity of EGaln nanocapsules was evaluated through *in vitro* cytotoxicity tests on HeLa cells and compared with other nanomaterials. Studies have shown that cells exhibit over 90% vitality at all concentrations, while the cell viability of other nanomaterials decreases with increasing concentrations (CHECHETKA et al., 2017). Thus, Ga-based liquid metals are one of the most promising materials for fabricating soft electronics, because they not only have excellent electrical conductivity, thermal conductivity, and stretchability, but also has biocompatibility.

**FIGURE 1:**
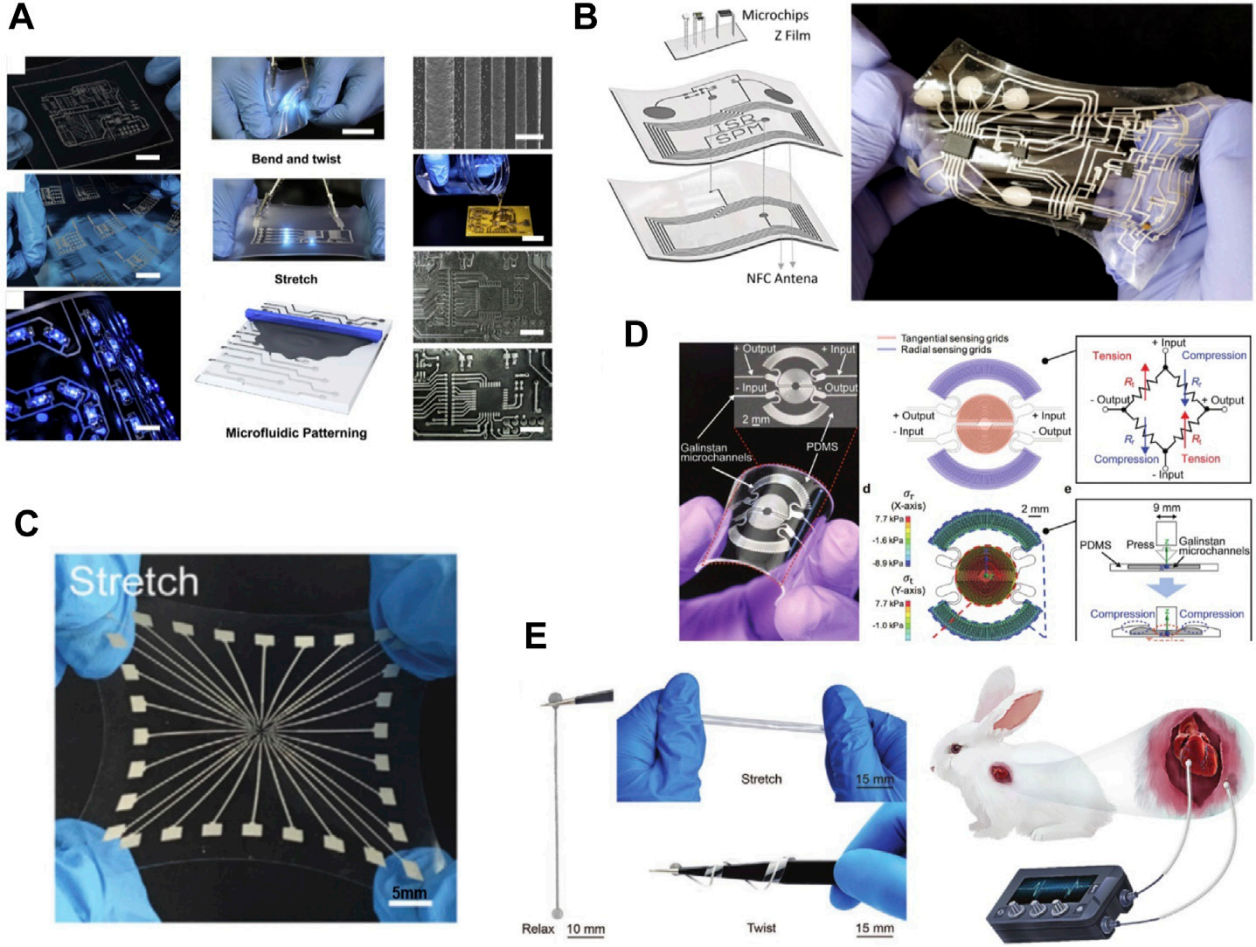
Applications of liquid metals in soft electronics. **(A)** Printing for making stretchable conductor (TANG et al., 2018) **(B)** Printing liquid metal to realize multi-layer circuit (LOPES et al., 2021a) **(C)** Highly stretchable liquid metal electrode array for Electrophysiology (DONG et al., 2021a). **(D)** Pressure sensor made of liquid metal (GAO et al., 2017) **(E)** Soft pacemaker made of liquid metal (HANG et al., 2021).

Before the advent of soft electronics, liquid metals are particularly attractive because of their low melting points, so they were often used in coolant, dentures, thermometers, and phase change material (ZHU et al., 2016; LIU et al., 2022a; IREI, 2022). With the development of soft electronics, more applications are developed according to the different properties of liquid metals. Those applications include:

Stretchable conductors for interconnects: liquid metals have liquid-grade deformability and metal-grade electrical conductivity, which makes them stretchable interconnects/wires for connecting electronics. Thus, stretchable devices with multifunctional purposes can be realized when combined with liquid metal printing techniques (TANG et al., 2018; GUO et al., 2019; GUO et al., 2022; LEE et al., 2022) (Figure 1A). It is also possible to implement multi-layered stretchable circuits through vias (GREEN et al., 2019; LOPES et al., 2021a) (Figure 1B). The excellent electrical conductivity and stretchability of liquid metal also make it a candidate material for electrodes (Figure 1C). It should be noted that the stretchability of the stretchable devices is usually much lower than that of stretchable conductors due the limitation of the connection between stretchable conductors and the rigid electronics. For example, the stretchability of some stretchable conductors such as liquid metals and gold nanowires has been reported to be as high as 1,000% (ZHU et al., 2013; CHOI et al., 2018; TANG et al., 2019a), however, when such stretchable conductors are used to make stretchable devices, the stretchability is usually below 300% (LU et al., 2015b; TANG et al., 2020). That is because most reported stretchable devices are realized by connecting rigid electrical components with stretchable conductors to achieve stretchability. When the stretchable devices are deformed, electrical failures usually occur at the interfaces between the stretchable conductors and rigid electronic components (soft–rigid connections) due to the stress concentration (LOPES et al., 2021b; TANG et al., 2022b).

Stretchable Antennas: With excellent electrical conductivity and stretchability, the liquid metal can be patterned into stretchable antennas for wireless communication and wireless power supply for soft electronics (XIE et al., 2020). Electronic devices are becoming softer, thinner and more conformable to human organs due to the fast development of conformal sensors, electrodes, and interconnects. However, making the battery conform to the human body is still challenging. To obtain 100% conformal devices, antennas composed of liquid metal could replace batteries to power the device. Through liquid metal printing technology, liquid metal can be made into antennas of different shapes, and the performance of the antenna can be easily adjusted by the shape of the antenna (CHENG et al., 2009; KUBO et al., 2010; QUSBA et al., 2014). While supplying energy, some liquid metal antennas can also transmit data from sensors in the device. For example, liquid metal antenna can integrate with a liquid metal strain sensors and NFC (near-field-communication) chips to constitute a stretchable devices for monitoring various human motions in a purely wireless fashion (JEONG et al., 2017). When the liquid metal antennas are combined with potentiometric electrochemical sensors such as sodium ion electrodes, potassium ion electrodes and ion selective electrodes, the devices have the potential to wirelessly detect metabolites (glucose) and electrolytes in sweat (MOU et al., 2021; MOU et al., 2022). The liquid metal antennas can maintain high-quality factor (q > 20) under stretching (>200% uniaxial strain), twisting (180° twist), and bending deformation (3.0 mm radius of curvature) (YAMAGISHI et al., 2021). Designing the antenna as different wavy structures based on structural engineering can greatly improve the performance of antenna under externally applied tensile strain (ZHU et al., 2019).

Soft sensors: When the liquid metal is deformed, its electrical parameters such as resistance and capacitance will also change. After the substrate undergoes deformation, the distance between adjacent serpentine liquid metal circuits changes, which alters the conductive path in the circuit. For example, when stretching, the distance between adjacent serpentine circuits increases and the resistance increases. The corresponding change in resistance or capacitance can be utilized as strain, pressure and tactile sensors. The most common liquid metal based-sensor is a resistive strain sensor, usually realized by printing liquid metal in a serpentine shape. Patterning tracks with reduced line width is necessary to increase the output sensitivity of the soft sensors. Liquid metal strain sensors generally cannot distinguish in-plane strain from normal stress, that is, stretching a sensor often gives a similar signal to pressing the sensor. To solve this problem, the structural design of the strain sensor can turn the strain sensor into a pressure/tactile sensor that is only sensitive to pressure (Figure 1D) (GAO et al., 2017). In addition, some capacitive strain sensors are insensitive to normal stress (ZHANG et al., 2022c). Liquid metal has high conductivity, so liquid metal resistance sensors usually have a small initial resistance, and the initial resistance is usually between 0.1 and 100 Ω. Compared with strain sensors based on nano-materials (YAN et al., 2021; KUMARESAN et al., 2022), both the resistant and the capacitive liquid metal sensors have low gauge factor, varying between 0.1 and 10, which means that the liquid metal sensors usually has a low sensitivity and a large measurement range (usually 0%–100%) (BOLEY et al., 2014; HIRSCH et al., 2016; COOPER et al., 2017; JEONG et al., 2017; DEJACE et al., 2019a; WU et al., 2021). Thus, liquid metal sensors are very suitable for measuring human motions by monitoring the angels of different joints (SHENG et al., 2016; DEJACE et al., 2019b; ZHANG et al., 2022c).

Soft electrodes for electrophysiological measurements: The liquid metal has extremely low modules. When patterning on thin and soft substrates, liquid metal can serve as conformal electrodes for electrophysiological measurements (YU et al., 2013; LI et al., 2022b) such as ECG, EMG, and EEG (Figure 1C). To realize electrodes with better conformability, free-standing liquid metal electrodes can be a better choice. Electrodes can be printed both on planar and 3D complex surfaces (ZHANG et al., 2019).

Soft thermal management materials: Liquid metals have been widely used as thermal management materials in high-performance convective coolants, phase change materials and thermal interface materials, which are mainly benefitting from their intrinsic high thermal conductivity (WANG et al., 2022a). To achieve stretchable thermally conductive materials stretchable, liquid metals can be embedded into the host of elastomers as thermally conductive pathways. This composite material enables rapid heat dissipation and prevents heat from being concentrated on the wearable device (BARTLETT et al., 2017).

Implantable devices: The gallium-based liquid metals have good biocompatibility because the gallium are regarded to be non-toxic to mammal cells (LI et al., 2018a; KIM et al., 2018; WANG et al., 2018). The toxicity of the liquid metal is believed from the released gallium ions (MOSCHèNSCHWEIZER et al., 2001), but the concentration of the gallium ion released by the implantable devices fabricated by liquid metal is well below the toxicity threshold. For example, Liquid metal wire has good stretchability and can be used as a lead for soft pacemakers for correcting abnormal heart rates in a rabbit model. The liquid metal soft pacemakers can be absorbed over time in the body, avoiding secondary injury caused by the remaining lead wires permanently left in the body (HANG et al., 2021) (Figure 1E). They also found that the amounts of Ga and In the major organs of the rabbit model are much lower than the LD_50_ value and the LD_0_ value. The liquid metal can also integrate with tissue engineering blood vessels as electronic blood vessel to promote cell proliferation and achieve gene delivery through electroporation (CHENG et al., 2017b). Thus, gallium-based liquid metals have great potential in fabricating implantable devices.

Printed OLED and batteries: The liquid metals have potentials to fabricate fully printed OLEDs and batteries. The liquid metal can serve as electron injecting (negative) electrode in an OLED. When OLED is composed of liquid metal cathode, electroluminescent polymer (such as poly (2-methoxy-5-(3′,7′-dimethyloctyloxy)-1,4-phenylenevinylene) and Ru (bpy)3(ClO4)2) and transparent anode electrode material (such as ITO and PEDOT:PSS), it can emit light at a voltage of about 3 V. All the components of the OLED can be realized by printing (GAO and BARD, 2000; SU et al., 2022). Though printing, liquid metal (anode) and Ag_2_O (cathode) can also form fully printed soft batteries (COSTA et al., 2022), which makes fully printed wearable devices possible in the future.

### 2.2 Patterning liquid metals for soft electronics

Liquid metals need to be patterned before being made into soft electronics. Methods for patterning liquid metals can be divided into patterning bulky liquid metal and patterning liquid metal emulsions. The difference between the two methods is that the liquid metal pattern in the former method is conductive after printing, while the liquid metal pattern in the latter method needs to be sintered after patterning to be conductive.

Patterning bulky liquid metal includes inkjet printing, microfluidic channel method, spraying, and vapor deposition. Inkjet printing is an efficient and low-cost technology for depositing and patterning materials. Because inkjet printing technology can form patterned film without the mask. The inkjet printing device usually includes an ink cartridge and an inkjet head capable of accurately depositing the solution in the design area. Inkjet printing can precisely control the volume and position of liquid metal deposition. And it is pollution-free and has high material utilization rate. Thus, inkjet printing has the most commercial application prospects in the field of personalized printed electronics. However, the low resolution of inkjet printing limits its application. Because liquid metals have great surface tension, liquid metals are limited to printing on surfaces that can be wetted by liquid metals (LU et al., 2022). The wettability of the liquid metal to the substrate, the viscosity, the oxide film on the liquid metal, and the nozzle diameter are essential parameters that affect the resolution.

**FIGURE 2:**
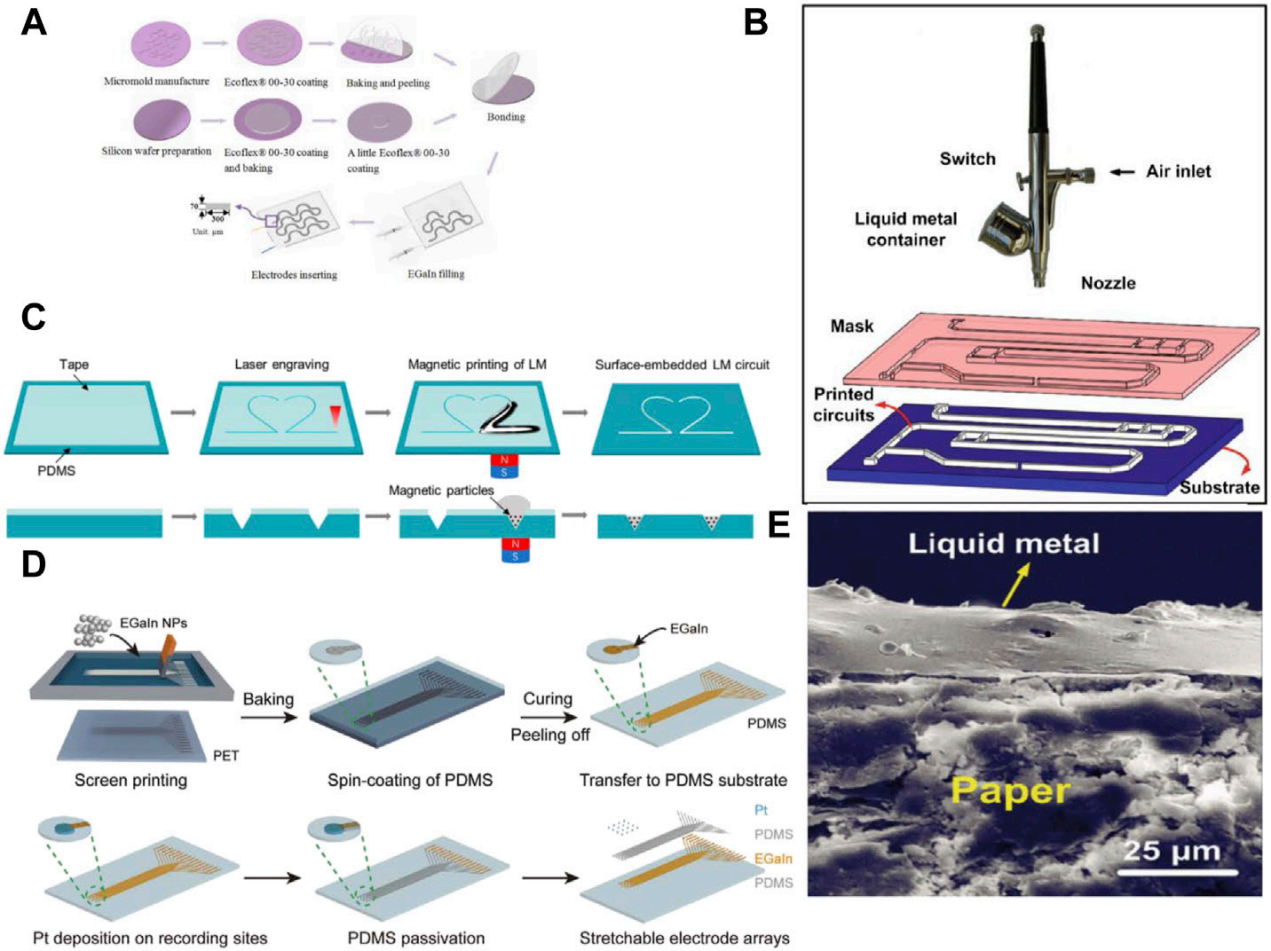
Patterning liquid metals for soft electronics. **(A)** Patterning of liquid metals by microfluidic (CHEN et al., 2020a) **(B)** Spray patterning of liquid metal and SEM images of spray on paper (ZHANG et al., 2013) **(C)** Printing liquid metals circuits by magnetic printing (ZHANG et al., 2022e) **(D)** Patterned liquid metal circuit by screen printing (DONG et al., 2021b) **(E)** Cross-section of the paper-liquid metal interface (ZHANG et al., 2013).

Liquid metals patterned by microfluidic channels method usually have higher resolutions than inkjet printing, and it is reported that the minimum line width of liquid metal patterned by microfluidic channel can reach 0.5–10 μm (PAN et al., 2018; AN et al., 2022). Briefly, the researchers fabricated the microfluidic channel based on silicones by soft photolithography, and then injected the liquid metal into the microfluidic channel by injection syringe (ZHANG et al., 2022d). Researchers fill the microchannel with liquid metal through electrochemical method, which avoids the influence of bubbles when injecting LM into the microfluidic channel (LI et al., 2022c). Soft sensors fabricated by microfluidic methods can accurately identify deformation, effectively reduce the hysteresis of the sensor, significantly reduce the error in the measurement process, reduce hysteresis and improve stretchability (CHEN et al., 2020a) (Figure 2A). The conductor produced using this method has a tensile strain of up to 200%. However, microfluidic channels can only be used to print continuous patterns of connected inlets and outlets due to the limitation of injection. In addition, the liquid metal requires greater injection pressure to fill the fine channels, and air also remains inside the micro-channels, which may lead to leakage and device failure.

Liquid metals have good liquidity and can be loaded into a spray gun (airbrush) for spray coating. Atomizing droplets of liquid metal rapidly oxidize in the air, which can significantly increase the adhesion of liquid metal. Thus, bulky liquid metal can be patterned in almost all kinds of substrates through masks (Figure 2B) (ZHANG et al., 2013; GUO et al., 2014a). The resolution of liquid metal patterns usually depend on the masks, and the line width for spray printing can reach 100 μm (GUO et al., 2014b). Microfluidic channels from soft lithography are used to achieve liquid metal patterns with high resolution (REN et al., 2019). Liquid metals are spraying on the PDMS substrate with the microfluidic grooves, and then the liquid metal outside the grooves is removed. After sealing the grooves with another layer of PDMS by ionic bonding, microfluidic channels filled with liquid metals are achieved.

Vapor deposition is a method of reactive synthesis of coatings or nanomaterials on the surface of a substrate, and is the most widely used technique in the semiconductor industry to deposit various materials, including a wide range of insulating materials, most metals and metal alloys. Two or more gaseous raw materials are introduced into a reaction chamber and then chemically react with each other to form a new material that is deposited on the wafer surface. At room temperature, LM has low vapor pressure (LIU et al., 2012). Heat Ga under a vacuum to obtain hot metal steam, and then condense on the receiving substrates, usually gold and copper substrates (HIRSCH et al., 2019), to form LM film. This approach allows for precise control of the deposited LM quantity and prevents the formation of the oxide skin. Liquid metal patterns from vapor deposition usually have high resolution (∼4.5 μm), which has potential for developing transparent conductors based on liquid metals (PAN et al., 2018). Moreover, a stretchable network of liquid metal conductors can be made on an elastic sponge by physical deposition, put a sponge mixed with styrene-isoprene-styrene (SIS) and salicylic acid in a thermal evaporator, then deposit liquid metal to produce a porous network of conductive sponges.

Usually, to obtain gallium-based liquid metal emulsions, large pieces of liquid metal need to be dispersed in solution through vigorous physical stirring such as ultrasonic treatment, high-speed stirring, and shearing. Compared with patterning bulky liquid metal, liquid metal emulsions can be printed onto the desirable substrates without being limited by the huge surface tension of the liquid metals. We can adjust the printability and stability of the liquid metal emulsion by adding additives such as surfactant, thickener, and special polymers (LOPES et al., 2021c; WANG et al., 2022b; JO et al., 2022), so that they can be successfully printed on different substrates.

Screen printing is a highly efficient method for patterning liquid metal emulsions, which takes the screen-printing plate with the pattern as the stencil. When printing the liquid metal emulsions, we pour ink into one end of the screen-printing plate, and apply a certain pressure to the ink part on the screen-printing plate with a scraper, and moving towards the other end at a constant speed, and the ink is squeezed from the mesh of the pattern to the substrate by the scraper during the movement (Figure 2C) (ZHANG et al., 2022e). The pattern resolution depends on the stencil fineness. The stencil thickness needs to be reduced to obtain high-resolution patterns, but the thinner the stencil, the more fragile it becomes (KIM and HONE, 2017). Although screen printing technology is usually used to print patterns with a larger width, liquid metal with a line width of 100 microns can also be printed by optimizing the screen printing process (DONG et al., 2021b). Screen printing can also be combined with spray coating, which can quickly spray liquid metal onto substrates and stencils over large areas (REN et al., 2019). However, the surface and edges of the pattern formed are usually not flat, and the liquid metal can remain on surfaces beyond the intended pattern.

Liquid metal emulsions can also be patterned by inkjet printers. Liquid metal emulsions are deposited onto substrate by a digital computer-controlled printer (BOLEY et al., 2015; ZHOU et al., 2020), allowing precise control of the amount of liquid metal deposited and improving printing resolution in the sub-micron range (DONG et al., 2021c; CHO et al., 2022; LEMARCHAND et al., 2022). Liquid metal with a resolution of up to 90 μm can be achieved by Ink jet printing (ZHOU et al., 2020). Compared with bulk liquid metal inkjet printing, liquid metal emulsion inkjet printing is not limited by the wettability of liquid metal, and there are more choices of substrates that can be printed.

Liquid metal can be patterned with magnetic fields. Adding magnetic particles to liquid metal can make liquid metal produce a magnetic response, and we can use magnets to control the patterning of liquid metal (Figure 2D) (DONG et al., 2021b). Magnetic printing can overcome the high surface tension of liquid metal and realize patterned liquid metal. The locomotion and morphological manipulation of the magnetic Liquid metal droplets can also be realized using arrays of electromagnets (LI et al., 2020a).

**FIGURE 3:**
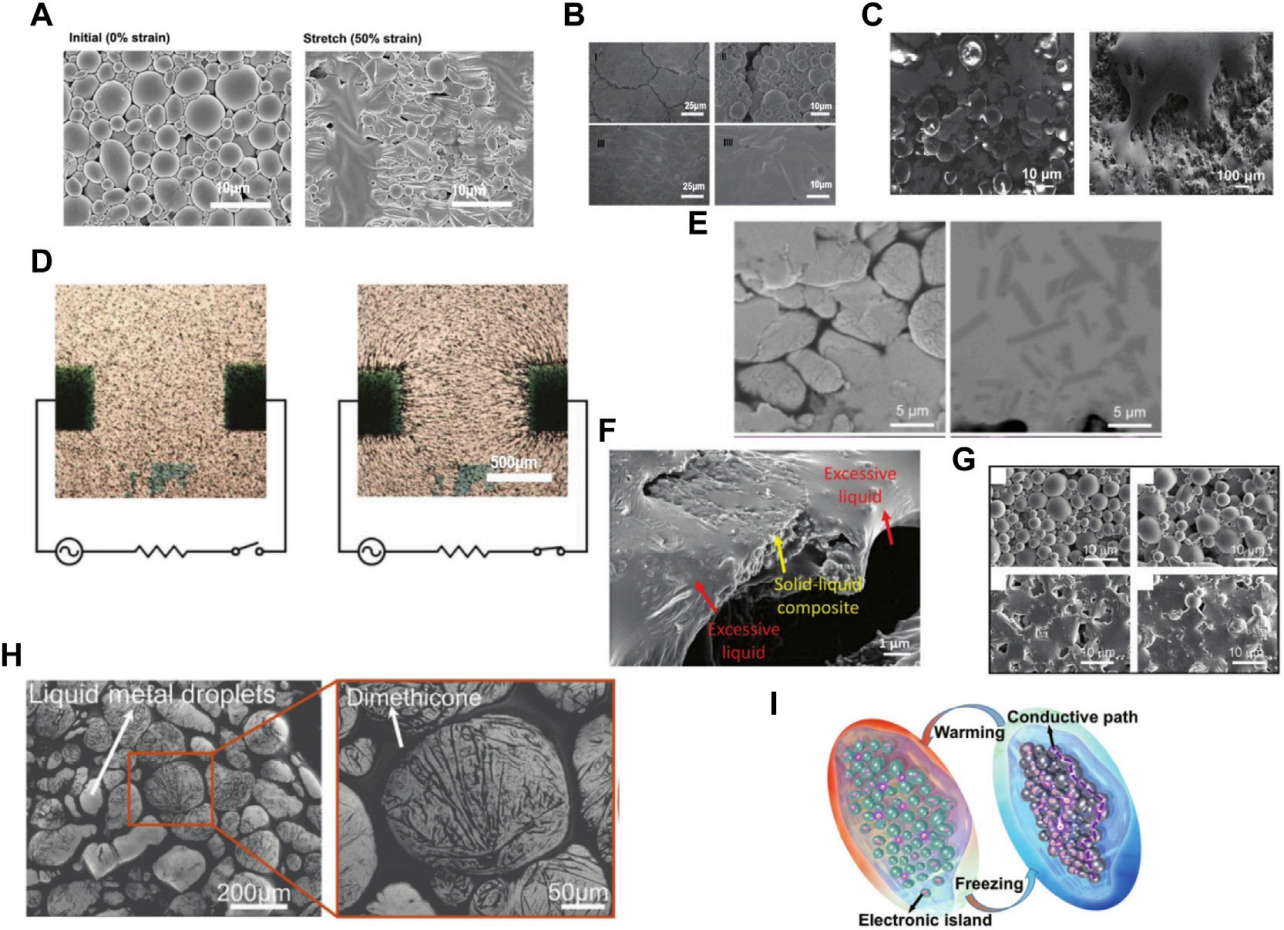
Sintered liquid metal **(A)** Strain sintered liquid metal (TANG et al., 2019b) **(B)** Pressure sintered liquid metal (LIN et al., 2015). **(C)** Heated sintered liquid metal (NIU et al., 2022). **(D)** Dielectrophoresis sintering of liquid metal (KRISNADI et al., 2020). **(E)** Chemical sintering of liquid metal (LI et al., 2020b) **(F)** SEM image of laser sintering (DENG and CHENG, 2019) **(G)** SEM characterization of the LM after different wet-dry cycles (TANG et al., 2020) **(H)** Pictures of liquid metal microdrops show that the liquid metal droplets cannot contact each other at room temperature (CHEN et al., 2019) **(I)** Schematic diagram of the conductor-insulation transition of the materials (CHEN et al., 2019).

Compared with patterns consisting of conductive bulky liquid metals (Figure 2E), patterns from liquid metal emulsions are composed of liquid metal particles that need to be sintered to be conductive. It should be noted that the pattern composed of liquid metal particles is not electrically conductive after printing, because there is an insulating oxide film on the surface of gallium-based liquid metal particles, which must be broken by the external stimulus to form conductive paths (LIN et al., 2017b). This process is also called sinter the liquid metal particles. Many external stimulus can sinter the liquid metal particles and make the pattern conductive, which include strain (Figure 3A) (TANG et al., 2019b), pressure (Figure 3B) (LIN et al., 2015), thermal sintering (Figure 3C) (NIU et al., 2022), dielectrophoresis (Figure 3D) (KRISNADI et al., 2020), chemical sintering (Figure 3E) (LI et al., 2020b), laser irradiation (Figure 3F) (DENG and CHENG, 2019), humidity (Figure 3G) (TANG et al., 2020) and freezing sintering (Figures 3H, I) (CHEN et al., 2019). For example, strain and pressure can sinter the patterns composed of liquid metal particles. Before the strain/pressure is applied, the particles are complete and isolated by the oxide layer. When the liquid metal particles are subjected to strain/pressure, the liquid metal particles will break and merge to form conductive ways. Thermal expansion microspheres are added for thermal sintering of liquid metal particles (NIU et al., 2022). After printing, LM is sintered into a conductive path through mechanical pressure caused by expansion of the microspheres after heating. Liquid metal particles can be sintered in dielectrophoresis. In a non-uniform electric field, particles suspended in the dielectric medium are polarized and subjected to a force, and this phenomenon is referred to as dielectrophoresis. The resulting dielectrophoretic force depends on the position of these particles in the electric field and the relative polarizability of the particles and the medium (LUMSDON and SCOTT, 2005). Thus, dielectrophoresis can be used to assemble, align, and sinter liquid metal droplets in uncured PDMS to form conductive paths (KRISNADI et al., 2020). Chemical sintering is to expose the liquid metal to acid smoke for a short time to remove the surface passivation oxide (LI et al., 2020b). This sintering method requires adding additional Cu to the liquid metal ink. After exposure to the acid smoke, the oxide layer of the liquid metal particles will dissolve and the liquid metal diffuses on the Cu nanosheet and forms a fully connected liquid metal layer. There are two possible mechanisms for laser-irradiated liquid metal sintering (DENG and CHENG, 2019). One is thermal cracking, and the other is evaporation. The liquid metal is heated by pulsed laser irradiation. The ultra-fast laser pulse will cause the rapid thermal expansion of the liquid core, which will generate tension on the solid shell. When the stress exceeds the critical value, the liquid metal shell will break and form a conductive path. Laser irradiates liquid metal and rapidly heats it above its vapor point, and the core material evaporates or ablates. The vapor radiates from the metal core, and disrupts the oxide shell. The vapor is then rapidly cooled in air or on the substrate and forms metal nanoparticles. In humidity sintering, the hygroscopic polymer should be added to the liquid metal ink, because the hygroscopic polymer on the liquid metal particles will shrink when the humidity changes from high to low. And the squeezed liquid metal particles will break and form a conductive path between the particles. Low temperature can also sinter the liquid metal particles since the liquid metal droplets expand as they freeze, decreasing the distance between the liquid metal droplets. Finally, the expanded droplets will contact and form a conductive path.

## 3 The spinning technologies for soft electronics

### 3.1 Electrospinning

**FIGURE 4:**
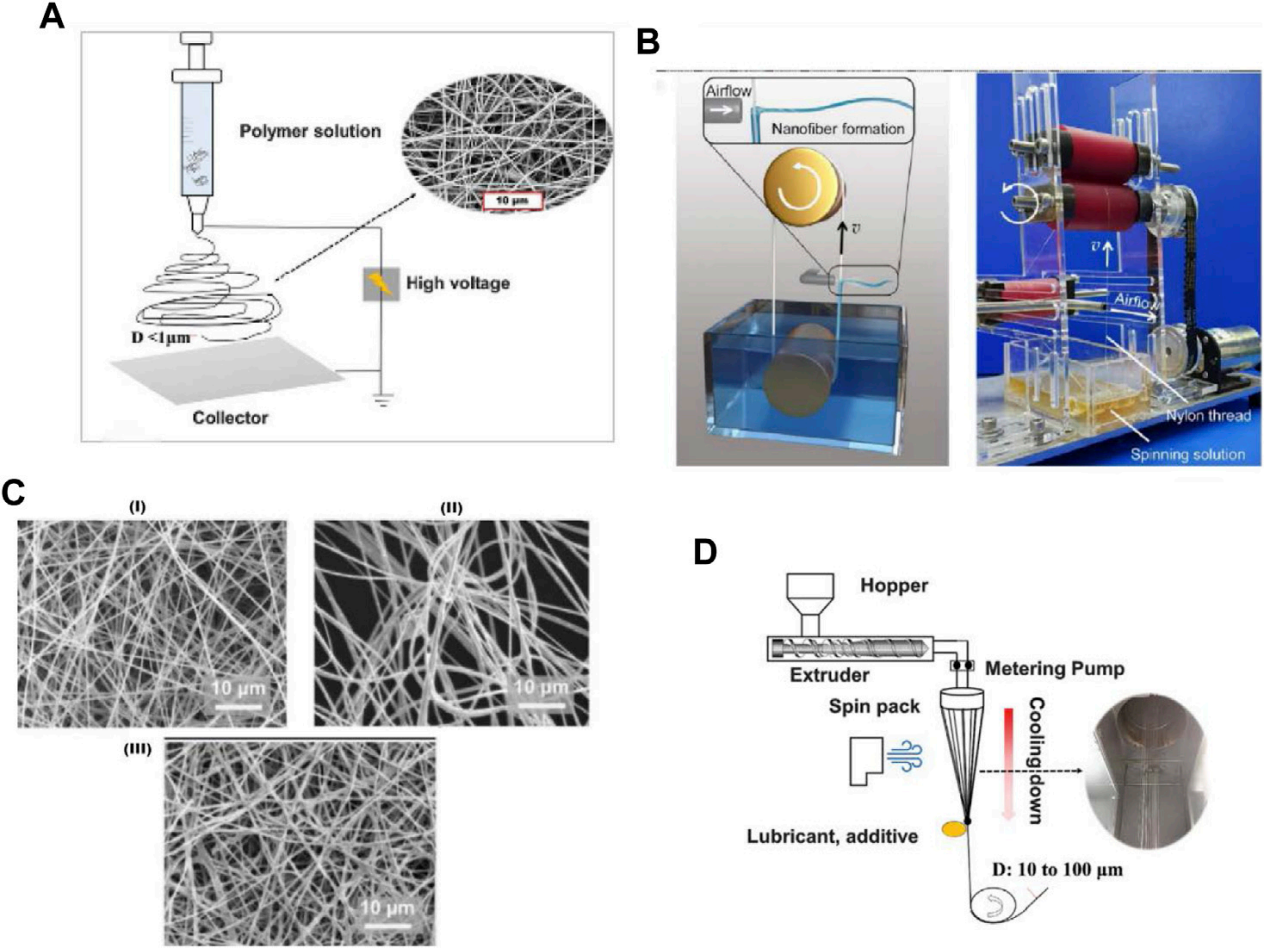
Schematic diagram of spinning equipment. **(A)** Schematic diagram of electrospinning device (ROSTAMITABAR et al., 2021) **(B)** Needle free air jet spinning with nylon rope instead of syringe (LI et al., 2022d) **(C)** Morphology of nanofibers prepared by different spinning methods (CAO et al., 2021) (I) Electrospinning and air jet spinning (II) Air jet spinning (III) Electrospinning **(D)** Schematic diagram of melt spinning (ROSTAMITABAR et al., 2021).

The earliest electrospinning may date back to 1934, in which Formalas developed an experimental apparatus for preparing polymer fibers by electrostatic forces (XUE et al., 2019). By this device, it is feasible to fabricate micrometer or nanoscale fibers in the presence of electric field forces with polymer solutions. An electrospinning device usually constitutes a high-voltage power supply and an injection pump. The positive cathode of a high-voltage power supply is connected to the spinning nozzle, and the negative cathode is connected to the receiver. The polymer solution is charged and ejected at the nozzle, and then shoots to the receiver and solidifies into fiber under the electric field force (Figure 4A) (ROSTAMITABAR et al., 2021). Compared with the melt spinning, the application of electrospinning materials is more extensive, making it possible to spin polymers that are not resistant to high temperatures such as natural polymers, fibroin protein, etc (HAN et al., 2022). In addition, electrospinning is usually performed at room temperature, which allows it to make drug-loaded or natural polymer fibers that are sensitive to high temperatures. For example, researchers used the electrospun poly (ε-caprolactone) and poly (dl-lactide-co-glycolide) membrane as the inner and outer layers of tissue-engineered blood vessels, respectively. *In vivo* observation and *in vitro* experiments show that this kind of blood vessel has good performance in shape maintenance and structural remodeling, which can approximately simulate natural blood vessels, paving the way for making biodegradable artificial blood vessels (CHENG et al., 2017b). Electric field is a significant parameter in electrospinning, irregular distribution of electric field strength can lead to irregular distribution of nanofiber, which reduces the efficiency of electrospinning process and strength of the fibers (SMOLKA et al., 2022). The temperature and humidity of the spinning environment can affect the diameter and shape of the fibers. The environment with high temperature and low humidity can make the faster evaporation of the spinning solvent, as is beneficial to facilitate the fiber formation. For different macromolecule polymers, the environment has a diverse effect on the fiber. For instance, the higher the humidity is, the larger the average diameter of chitosan nanofibers becomes, while the smaller the diameter of polyvinyl pyrrolidone nanofibers gets (SZEWCZYK and STACHEWICZ, 2020). It was found that the average diameter of silk fibroin/poval nanofibers decreases with increasing humidity, so that different environmental parameters should be controlled for different polymer materials to obtain an ideal fiber morphology (PARK and UM, 2021). Electrospinning fibers can also form beaded structures, affecting the stretchability of the fiber membrane. The main causes of beaded structures include: the concentration of the polymer solution, the electrospinning voltage, and the molecular weight of the polymer. At lower concentrations, the fibers containing more bead-string structures will be gained. As the concentration increases, the resulting fibers are gradually uniform, and when the concentration is further increased, the spiral nanoribbons will be obtained (KOSKI et al., 2004; EDA and SHIVKUMAR, 2007). The low molecular weight of the polymer creates resistance to the jet stretching flow, so electrospun nanofibers tend to form beaded structures. When the molecular weight reaches the appropriate range, jet stretching flow will stabilize and form a uniform nanofiber. When the molecular weight is overly high, the diameter of the fiber and the fiber interval further increases, and the shape of the fiber cross section gradually changes from round to flat shape (MEDEIROS et al., 2022). On the condition that the applied voltage exceeds a certain threshold value, a stable polymer solution jet will take shape. The researchers found that a higher voltage will make the fiber diameter finer, which is propitious to the fabrication of nanoscale fibers (PARHAM et al., 2020). However, if the voltage continues to increase, the fiber diameters will be larger and evenness will be poorer, forming beaded or string-beaded nanofibers (LU et al., 2021).

Electrospinning technology have been widely used to fabricate nanoscale fibers. The advantages of electrospinned nanofiber mesh include large surface area to volume ratio and high porosity (YAN et al., 2019). In recent years, electrospinning has been commonly used to make nanofiber scaffolds. Compared to traditional scaffolds, cells are more likely to penetrate and migrate on electrospinned scaffolds (LIN et al., 2020). The cellulose scaffold made by electrospinning has a porosity of up to 94%. Compared with other scaffolds, the scaffold significantly increased cell proliferation after 7 days of cell inoculation (KI et al., 2008). The different electrospinning materials will also affect the porosity of the fiber mesh. The scaffolds with low (76%), medium (83%) and high (90%) porosity were prepared using polyethylene oxide. Research shows that fiber scaffolds with high porosity are more suitable for cell migration and proliferation because of lower fiber density (VOORNEVELD et al., 2017). The polyurethane fiber film made by electrospinning exhibits a tensile strain of 372.4% and a water contact angle of 137.1°, exhibiting excellent waterproof and breathable properties, making it an ideal candidate substrate for skin electronics (ZHOU et al., 2021a). A polyurethane solution containing MXene was electrospun to produce conductive yarns with a tensile strain of up to 253% and a conductivity of 1195 S/cm, which showed potential in body motion monitoring (LEVITT et al., 2020). Electrospinning can also make rigid materials into stretchable materials, and ceramic nanofibers produced by electrospinning have a tensile strain of up to 100% (CHENG et al., 2022).

### 3.2 Air-jet spinning

In 2009, Medeiros proposed air-jet spinning, which is a new spinning method in recent years, producing polylactic acid and polystyrene fibers and studying the influence of airflow on fiber morphology through air-jet spinning technology (GAO et al., 2021). In this process, the solution is extruded from the needle tip to eject liquid jet in the direction of the gas flow. The gas flow not only acts as a driver but also effectively assists in the evaporation of the solvent, leaving uniform fibers. This technology has applied to construct a spirally arranged cardiomyocyte scaffold, on which cardiomyocytes were successfully cultured and beat (CHANG et al., 2022). Spinning needle-based technology is an established method to produce fibers efficiently, but there is a risk of needle clogging due to the rapid evaporation of the solvent. Researcher developed needleless air-jet spinning technology, which can strengthen the shear stress of liquid jet through stronger spinning airflow, enhance the drawing effect and solvent evaporation significantly during spinning (LI et al., 2022d) (Figure 4B). Air-jet spinning materials are more versatile than melt spinning and electrospinning, which is independent of electrical conductivity and thermoplasticity, so they have a wide range of applications. However, the fibers produced by air-jet spinning have loose and flexible morphology and poor mechanical properties (GRANADOS-HERNáNDEZ et al., 2018).

Air-jet spinning can be combined with electrospinning. For example, in a spinning device, the cathode of the high-voltage power supply is connected to the roll collector, and the anode is connected to the spinning needle to provide an electrostatic field. The solution is blown out through both high-speed airflow and the electrostatic field to obtain nanofibers. The introduction of an electrostatic field in air-jet spinning can effectively enhance the traction and stretching effect of the solution, which is beneficial for obtaining uniform fibers. The researchers have developed a flame-retardant rayon/graphene nano-ion electronic skin by electrospinning and air jet spinning technology (CAO et al., 2021), which has the advantages of electrospinning and air-jet spinning. The produced fiber is more uniform, unpliant, strong stretchability and high production efficiency, and has bright prospects in artificial skin protection (Figure 4C). The looser fibers produced by air-jet spinning are more suitable for the production of cell scaffolds than the denser fibers produced by electrospinning, which will affect the penetration of cells. Researcher prepared polycaprolactone cell scaffolds containing diamond nanosheets by air-jet spinning, which not only improves the mechanical strength of scaffolds, but also promotes the cell proliferation, and can be extended to cell scaffolds required by various tissue engineering (AUGUSTINE et al., 2023). Tests showed that it is easier to form cylindrical uniform fibers by increasing the concentration of solute in the solution; while the low-concentration polymer solution will produce beaded structure and reduce the tensile properties of the fiber membrane although the fiber membrane will become thinner (YANG et al., 2023). The air-jet spinning technology with high efficiency, short preparation time and low solution requirement will be beneficial to the large-scale production of nanofibers and realize industrial production.

Air-jet spinning can produce fibers with high porosity, and the diameter of fibers usually varies from nanometer to micrometer. Compared with electrospinning, air-jet spinning has a higher yield and has the potential to achieve mass production (GAO et al., 2021). For example, polyethylene oxide and polyvinylpyrrolidone fiber membranes produced by a specially designed jet spinning method usually have fiber diameters ranging from tens of nanometers to several microns (BENAVIDES et al., 2012), and the production speed is 10–20 times that of a single electrospinning (usually range from 0.1–1.0 g/h per jet) (LI and XIA, 2004; GREINER and WENDORFF, 2007). The polyvinyl acetate fiber membrane containing titanium dioxide produced by airbrush spraying apparatus has a porosity of up to 93%, with an average pore size of 1.58–5.12 μm (ABDAL-HAY et al., 2015). Through the improvement of spinning equipment, the production rate can be further increased. The hyaluronic acid scaffold made by immersion rotary jet spinning has a porosity of up to 95% and a diameter from 500 nm to 3 μm. And the manufacturing throughput of the air-jet spinning reaches about 1 g/min which is much higher than electrospinning. The scaffold accelerates granulation tissue formation, blood vessel formation, and re-epithelialization, promoting wound closure (CHANTRE et al., 2019).

### 3.3 Melt spinning

Melt spinning is a kind of method of producing nanofibers, and it uses molten polymers as the spinning solution. In the past, melt spinning has become an essential method for manufacturing nanofibers. It is first, heating Polymer to the melting point. Then, the molten polymer is extruded from the spinning head (ROSTAMITABAR et al., 2021) (Figure 4D). The most commonly used materials for melt-spinning are polyamides (HABERKORN et al., 1993), polyesters (ALATAWI et al., 2023) and polyolefins (KIM et al., 2021). The basic requirement for melt spinning is that polymer does not decompose when melted at relative high temperature. Thus the melt-spinning technology does not apply to some natural polymers or fibers containing medications, especially when medications need to be directly added to the spinning material, which limits its application in the medical field.

In melt spinning, some additives are usually added to the melt to facilitate processing or improve the function of fibers. There are three basic types of additives: Processing aids (HUFENUS et al., 2020a; ZHANG et al., 2023), Enhancing additives (MAQSOOD et al., 2019; PENG et al., 2019; HUFENUS et al., 2020a), and Functional additives (HUFENUS et al., 2020b; BELKHIR et al., 2021; GOLL et al., 2021). Processing aids make nanofibers easier to form, reinforcing additives improve mechanical properties of nanofibers, while functional additives expand the properties of nanofibers. Moisture can strongly influence processability and cause the degradation of polymers in extrusion. Because melt hydrolysis leads to lower molecular weight, making fibers difficult to form. Thus, drying polymers is critical to the Melt spinning (ROSENBAUM et al., 2022). For some polymer materials, the hydrolysis of polymer materials will produce harmful substances. For example, the hydrolyzed PVDF melt will have highly corrosive and toxic hydrogen fluoride. Improper setting of process parameters in the melt spinning process will lead to the formation of a spherical structure in the fiber film, which will make the fiber brittle and not conducive to stretching (LI et al., 2018b). The construction of this structure mainly depends on the feeding rate and quenching rate at the nozzle. A higher feeding rate will make it easier to form this bead structure.

Melt spinning can also produce substrates with pores, but compared to fiber substrates made by other methods, the fiber substrates made by melt spinning have smaller porosity and larger fiber diameters (KRIEGEL et al., 2008). For example, the average porosity of polyvinyl chloride fiber membrane prepared by melt spinning is approximately 60% (LU et al., 2019). The porosity of the polycaprolactone scaffold made by melt spinning is 75% (CHUNG et al., 2010), and the diameters of both fibers range from several to hundreds of microns. After melt spinning, immersion coating and freeze-drying can not only improve the stability of the cell scaffold, but also increase the porosity to 97% (BUTTAFOCO et al., 2006).

## 4 Fabrication of liquid metal fibers

Fiber is the basic unit of the nanofibrous membrane. We can achieve liquid metal-based breathable electronics by weaving liquid metal fibers or directly printing liquid metal on spun films. Thus, it is usually necessary to prepare liquid metal fibers (LMFs) before weaving. There are several strategies for fabricating liquid metal fibers including injecting LM into a hollow fiber, dip coating, 3D printing, electrospinning, and biological manufacturing (Table 1).

**TABLE 1:** Structure, fabrication, and applications of liquid metal fibers.

LMF core	LMF sheath	Manufacturing method	Stretchability	Applications	Diameter
EGaIn (ZHU et al., 2013)	SEBS resin	Injection	700%	Conductive fiber	∼240 μm
EGaIn (COOPER et al., 2017)	Hytrel	Injection	150%	Torsion, strain, Touch sensor	∼800 μm
EGaIn (LAI et al., 2021)	SEBS resin	Injection	>650%	Nanogenerator, Dynamic monitoring sensor	2000 μm
Ga-In-Sn-Zn alloy (YU et al., 2020)	PU	Injection	480%	Dynamic force sensor, motion indicator	250 μm
EGaIn (ZHENG et al., 2013)	-	3D-printing	-	3D conductor structure, flexible antenna	510 μm
PU (MI et al., 2021)	EGaIn/	Dip-coating	up to 400%	Electroluminescent fibers, conductive fibers	∼600 μm
Polymethacrylates coated PU (CHEN et al., 2020b)	EGaIn	Dip-coating	500%	Conductive fiber	∼200 μm
EGaIn (MA et al., 2022)	PU	Dip-coating	1,273%	3D stretchable conductors, sensors	108 μm
Polymethacrylates coated PU (GUO et al., 2020)	EGaIn + Cu particles	Coating	300%	Conductive fiber, artificial muscle	∼112 μm
Ag coated-SBS (ZHUANG et al., 2021)	EGaInSn	Electrospinning + inkjet printing	2,500%	Stretchable circuits	8.6 μm
LM particles (NING et al., 2023)	PU	Coaxial wet spinning	232%	Energy harvesting and self-powered sensing	180 μm
LM particles + TPU (LIU et al., 2022b)	CNT + AgNW	Wet-spinning + dip-coating	500%	Wearable sensor	∼6–100 μm
Ga-In-Sn (YU et al., 2022)	PU	Coaxial wet-spinning	373%	Wearable sensor, heater	∼1,000 μm
LM particles + PVDF (ZHENG et al., 2021)	PVDF + PEGDA	Coaxial wet-spinning	1,170%	Heater, self-powered sensing	∼270 μm
LM-silk fiber (GAO et al., 2022)	-	Silkworm feeding	70%	Wearable sensor	∼40–250 nm
PVDF-LM particles (YU et al., 2018)	-	Electrospinning	∼30%	Nanogenerators	∼100 nm
Low melting point alloy (T_m_ = 62 °C) (NING et al., 2023)	Silicone rubber	Injection	400%	Variable Stiffness Fiber	250 μm
LM particles + Bisphenol-A epoxy (PENG et al., 2021)	-	Mold -	-	Temperature-sensitive conductors	89 μm
LM particles + PDMS (LIU et al., 2020)	-	Injection	∼1,400	Shape memory conductors, temperature electrical switches	1,100 μm
EGaIn + Fe particles (HONG et al., 2021)	SEBS resin	Injection	∼600%	Electrical switches for remote magnetic actuation	1,400 μm

**FIGURE 5:**
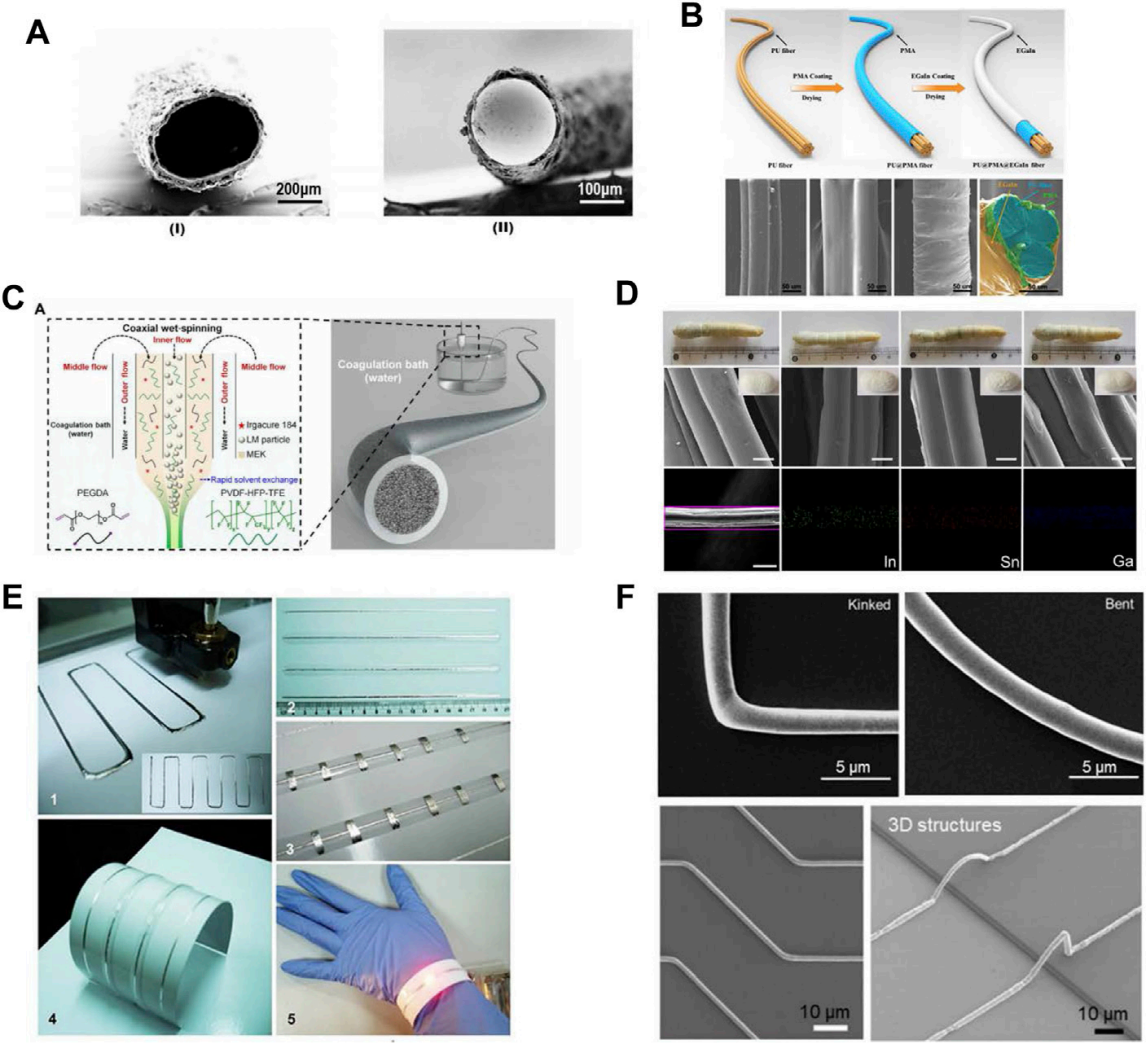
Manufacturing of liquid metal fibers. **(A)** Hollow fiber and injected liquid metal enter hollow fiber to produce liquid metal fiber (YU et al., 2020) **(B)** Schematic for the preparation of LM-coated fiber (CHEN et al., 2020b) **(C)** Schematic diagram of making core-shell liquid metal fiber by electrospinning (ZHENG et al., 2021) **(D)** Liquid metal feeding silkworm to obtain liquid metal fiber (GAO et al., 2022) **(E)** Schematic diagram of liquid metal fiber produced by 3D printing (ZHENG et al., 2013) **(F)** SEM of liquid metal fiber with 3D structure (PARK et al., 2019).

Injecting LM into a hollow fiber is one of the earliest reported strategies to fabricate LMFs. Injecting liquid metal into hollow a fiber by using a syringe can form a core-shell structure with a liquid metal core and polymer shell (COOPER et al., 2017; LAI et al., 2021). The LM-injected fibers usually have stretchable polymer shells (usually styrene resin) that can be stretched to strains of up to 800%, and maintains metallic conductivity due to the LM core. These LM-injected fibers are usually used as temperature, torsion, strain, and touch sensors, because external physical stimuli (deformation, temperature) induce changes in liquid metal resistance or capacitance. Also, those fibers can be used as a stretchable wire for earphones and battery chargers, with the same performance as standard components (ZHU et al., 2013). The mechanical measurement with or without liquid metal inside the fiber shows that the effect of liquid core on the mechanical properties of the fiber is negligible. The diameter of the LMF mainly depends on the inner diameter of the hollow shell, which ranges from several hundred micrometers to several millimeters. When the diameter is large, it is difficult for the internal LM to fill the hollow shell, resulting in uneven distribution of LM. Conversely, when the diameter is small, it is difficult for LM to inject extremely minuscule hollow fiber as the injection resistance increases dramatically. Using microfluidic technology (YU et al., 2020) (Figure 5A) or vacuum suction (LIN et al., 2017c), not only can the liquid metal be effectively injected into the fiber, but also the air trapped in the fiber can be reduced. For example, one inlet of the hollow fiber can be covered with LM, and the structure can be placed in a vacuum chamber to remove the air inside. After restoring the atmospheric pressure, the positive pressure gradient could quickly push the metal through the fiber.

LM can adhere to the surface of some fibers after dip coating. LMFs can be manufactured by immersing polymer fibers into LM or inks composed of LM particles, and LM/LM particles will directly adhere to the surface of the fiber through dip coating to form a liquid metal fiber. This method can apply to various fibers, such as polyurethane, hemp, and cotton (GUI et al., 2017; CHEN et al., 2020b; GUO et al., 2020). Using dip-coating method, highly stretchable fibers with superior stretchability and electrical conductivity (>100 S/cm when stretched to 500% strain) can be fabricated, which usually contains a polymer core, an intermediate modified layer to enhance the wettability of the liquid metal, and an outer liquid metal layer. Those fibers show excellent thermal stability, and the maximum operating temperature is close to 250°C. Those fibers can sever as wires for charging mobile phones, as sensors to detect the motion of the human body (CHEN et al., 2020b), and as light-emitting fibers (Figure 5B) that can be woven into glowing cloth (DONG et al., 2022). A study found that ultrasonic liquid metal treatment in fluid lactone will generate ring-opening polymerization. Through polymerization, liquid metal droplets are sealed in polyester shells and dry to a solid powder. Then, it is imported to thermoplastic composite by liquid casting and hot forming to produce liquid metal fiber. This method can improve the issue that liquid metals do not adhere easily to polymers (LI et al., 2020c). In addition, it has been reported that adding metallic particles such as nickel, iron, and copper through sufficient stirring generates more oxide within the LM, thus significantly enhancing the wettability and adhesion of the LM to the substrates. Thus, LM doped with copper particles can directly coat the fiber surface, and the resulting fiber exhibits desirable mechanical and electrical properties (GUO et al., 2020). Modifying the liquid metal particles with MXene can enhance the particles’ adhesion to the fiber. And the MXene can also bridge the adjacent particles to form conductive paths. Due to the addition of the MXene, the fibers also have electromagnetic interference shielding and joule heating properties (YI et al., 2022). The polymer core in the LMFs can be porous, for example, porous wires can be prepared after cutting SBS electrospinning mat into wires, and then LM can be coat can the porous wire to form a core-shell structure. The porous core of the LMFs can substantially improve the stretchability (∼2000%) and electrical stability of the LMF compared with the fiber with a nonporous core (ZHOU et al., 2021b).

Solid gallium wire can be easily made into a 3D helical structure. Coating polyurethane to the solid wire and liquefying the solid wires can retain the structure of the wire, thus forming stretchable conductors with 3D structures. The study shows that such conductors have a 1,273% breaking strain. The fiber diameter can be reduced by applying strain to the fibers during polyurethane curing (MA et al., 2022). The abrasion resistance and stability of LM-coated fibers during practical applications should be considered. After stretching, the highly oxidized LM are at risk of cracking, leading to electrical failure. When the LM-based conductive coating of fibers is exposed to the ambient environment, the coating easily adheres to any other contacted objects, leading to the contamination of neighboring objects and the loss of the LM coating. The LMF made by these methods can be directly woven into cloth to make breathable, soft devices. Those fabrics need to be strong, low-cost, wear-resistant, and washable before daily use, and thus we are still facing so challenges such as good encapsulation, surface modification, and mass-production of LMFs.

Electrospinning is a straightforward method for fabricating micro/nano fibers in the laboratory. However, due to the high conductivity of LM, it is difficult to charge it directly. Thus, using electrospinning technology to instantly produce ultra-thin LMF is still a challenge. Wet-spinning process can be a substitute for electrospinning. To obtain LMFs by wet-spinning, liquid metals need to incorporate different polymers to form composite spinning solutions composed of liquid metal and uncured polymers (LIU et al., 2022b). Coaxial wet spinning of liquid metal and polymer solution can form microfibers with core-sheath structure (usually liquid metal core and elastic polymer sheath). Coaxial electrospinning requires a coaxial needle. The inner channel of the needle is usually filled with liquid metal ink, and the outer channel of the needle is generally filled with elastic polymer solutions that constitute the sheath of the fiber after curing. A Recent study used LM as the inner channel and polyurethane polymer solution as the outer channel to produce fibers with high stretchability (up to 373%) and electrical conductivity (up to 3.4 × 10^6^ S/m) through a coaxial wet spinning process (YU et al., 2022). To further reduce the diameter of the fiber fabricated by coaxial spinning, a liquid metal composite consisting of liquid metal particles and polymer fillers can be used to fill the internal channels of the needle. The diameter of the fiber can reach about 270 μm, which is slightly larger than the hair. The fiber has a uniform surface and stable conductance, which can be easily woven into an everyday glove or fabric, acting as excellent joule heaters, electrothermochromic displays, and self-powered wearable sensors to monitor human activities (ZHENG et al., 2021) (Figure 5C).

The liquid metal is non-toxic and biocompatible, so it can also be mixed with silkworm feed to produce silk fiber by drip-feeding worms. The fiber produced by this method can significantly improve the tenacity of silk, and is an ideal material for making stretchable devices (GAO et al., 2022). Liquid metal-feeding worms produce silk containing liquid metal (Figure 5D). This kind of silk is conductive and easy to stretch. The silk containing LM obtained by feeding silkworms with LM may solve the problem that LM is not easy to charge during electrospinning, and also avoid the mutual interference of the spiral cones of the inner and outer fluids during coaxial spinning, and become an excellent electrospinning material (GAO et al., 2022).

Combining with 3D printing technology, liquid metal ink can be directly printed into liquid metal fiber (LMF) (ZHENG et al., 2013) (Figure 5E). It is reported that the minimum width of the 3D LMF reached 1.9 μm when printed through fine nozzle. Those LMFs can be used to develop ultra-fine, soft, and conductive interconnects for stretchable integrated circuit (PARK et al., 2019) (Figure 5F). Furthermore, they have the potential to be woven into textiles to be wearable devices after solidification of the LMF. The combination of LM and 3D printing technology reduces the etching process required by LMF manufacturing. It should be noted that LM ink usually needs pre-treatment to decrease the surface tension to the substrate. 3D printing technology is expected to promote the rapid prototyping of LM fiber. Although promising, the printed LM wire cannot be applied in stretchable electronics without proper substrate and encapsulation. Overall, integrating LM with 3D printing technology will great simplify the fabrication process of the LMF.

## 5 Liquid metal enabled soft electronics based on fibers

Electronics enabled by liquid metals usually have excellent stretchability. When porous substrates composed of fibers are adopted, such electronics will become air-permeable and conformal, allowing the electronics to adhere to the skin for a long time without causing redness and irritation. The liquid metal-enabled elastic electronics based on fibers can be achieved by weaving LMFs into smart textiles or patterning liquid metal directly on the electrospinning mats (ZHANG et al., 2020b; DONG et al., 2020; LAI et al., 2021).

**FIGURE 6:**
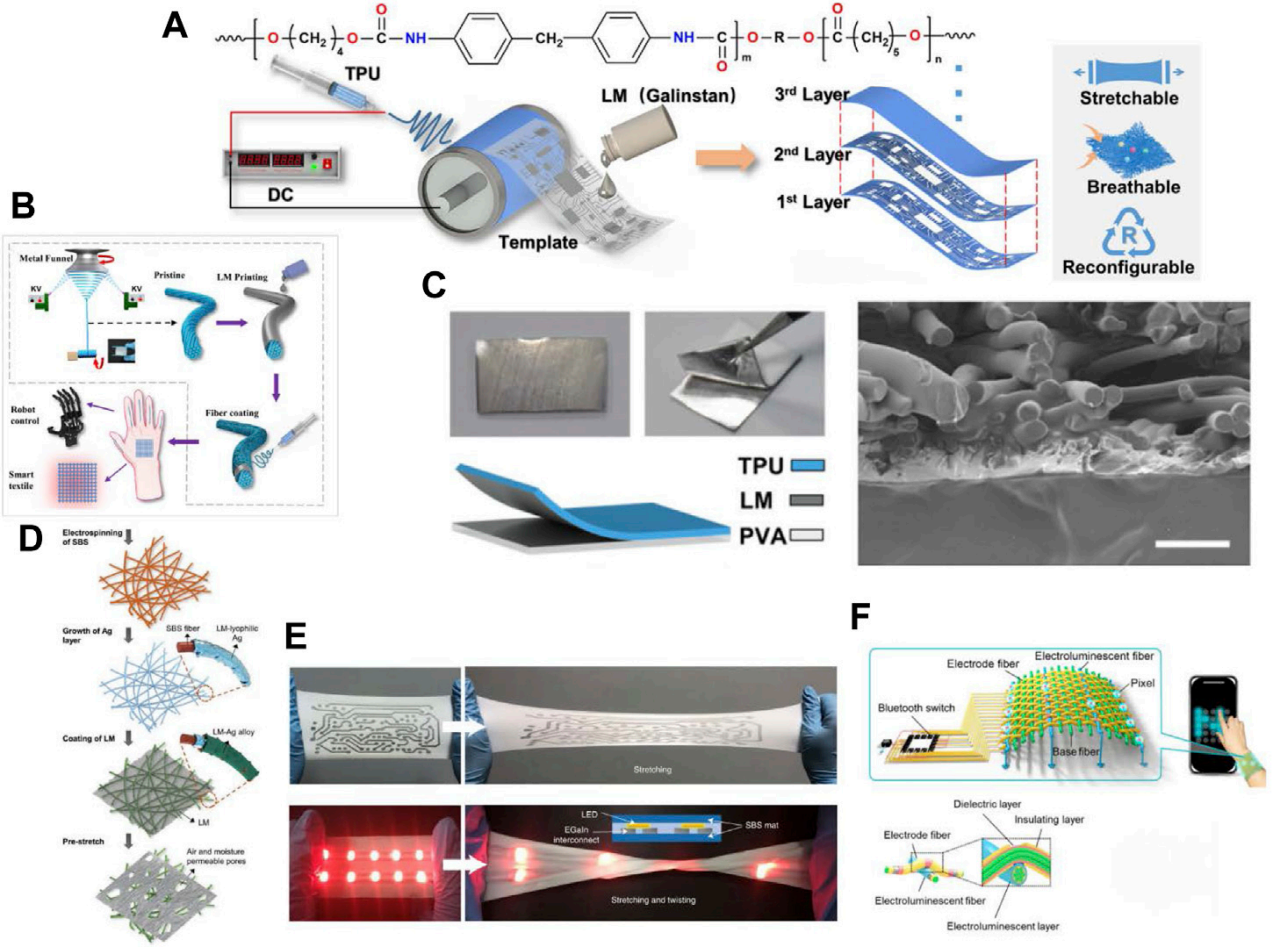
Liquid metal enabled elastic electronics based on fibers. **(A)** Sensor made of liquid metal printed on electrospinning substrate (WANG et al., 2021) **(B)** Making soft sensor by coating liquid metal on electrospun fiber (UZABAKIRIHO et al., 2022) **(C)** Stretchable adhesive liquid metal fiber mat (WANG et al., 2022d) **(D)** Composite electrode made of liquid metal fiber mat (ZHUANG et al., 2021) **(E)** Liquid metal fiber mat with LED (MA et al., 2021) **(F)** Liquid metal fiber that can emit light and image (MI et al., 2021).

The successful fabrication of nanoscale fibers has also lead to great advances in the soft electronics. It is reported that the high specific surface area of the nanofiber membrane is not only conducive to improving water permeability and air permeability, but also conducive to improving mechanical and electrical performances such as electrical conductivity (WANG et al., 2022c), stretchability, and sensitivity of sensors (TANG et al., 2022c). At present, electrospinning is an effective method to produce nanofibers, which can realize the production of fibers with different diameters and shapes by adjusting the parameters of electrospinning devices. Printing, spraying, coating and other methods are adopted to pattern liquid metal on the electrospinning mat, and liquid metal can be infiltrated into the fiber membrane to realize the conductive stretchable substrate. The nanofiber membrane prepared by electrospinning owns a unique network structure, and its deformation and fracture can change the conductive network and lead to a short-term change in resistance. With good encapsulation of liquid metal, sensors with high elasticity, high sensitivity, and good air permeability can also be achieved on the electrospinning substrates (WANG et al., 2021) (Figure 6A). Compared with the sensors printed on non-fibrous membranes, those printed on the fiber membrane exhibit higher sensitivity and elasticity, enabling them to respond quickly to micro-signals such as pulse, respiration, and voice. Compared with strain sensors based on the TPU/LM fibers (Figure 6B) (UZABAKIRIHO et al., 2022), those printed strain sensors have a straightforward fabrication process. Besides, the unique 3D porous network structures bring them excellent air permeability, so that they can fit onto the human skin surface more comfortably.

To achieve stretchable circuit on porous substrate, liquid metals can be directly printed on the TPU nanofiber membrane through masks. The rigid electronics are fixed on the stretchable substrate through a polyvinyl alcohol glue, thus ensuring the stability and reliability (WANG et al., 2022d) (Figure 6C) In order to obtain a stable connector suitable for connecting soft conductors and rigid components, researchers obtained a new material that can be used as conductive adhesive after uniformly mixing liquid metal with SBS solution by ultrasound. 11-mercaptoundecanoic acid was added into the solution to remove the oxide on the surface of liquid metal particles to make them conductive. And this material is also suitable for printing or casting soft conductive matrix (MOU et al., 2020). To enhance the wettability of nanofiber membranes by liquid metal, the researchers modified the nanofibers with silver nanoparticles to obtain the LM-superlyophilic mat, on which LM can be readily coated or printed. The liquid metal forms a network in the lateral and vertical directions in the nanofiber membrane, realizing the stretchable electronic equipment with high air permeability and high stretchability. The air permeability and moisture permeability of the fiber mat is higher than those of nylon cloth and medical patch by testing. The resistance of the fiber membrane changed by less than 25% after 25,000 tensile cycles at 60% strain, making it a good material for wearable devices (ZHUANG et al., 2021; PARK et al., 2012) (Figure 6D). Researchers also found that liquid metals can be easily coated or printed onto the poly (styrene-block-butadiene-block-styrene) (SBS) fiber mat, which offers simultaneously high permeability, stretchability, conductivity and electrical stability. When a liquid metal circuit is encapsulated with a second layer of SBS mat, the encapsulated device remains functional even after washing under water (MA et al., 2021) (Figure 6E).

The performance of liquid metal devices can also be tuned by changing the orientation of the fibers. A high-speed collection device is adopted to prepare a unidirectional fiber membrane, based on which a high-sensitivity sensor with unidirectional sensing can be produced by printing the liquid metal on the fiber membrane through screen printing (YANG et al., 2022b). In this way, a directional biaxial strain sensor can be made by placing two layers of fiber membranes orthogonally, and the magnitude and direction of the strain can be obtained by theoretical calculation. The directional sensing sensor fabricated by this method shows great potential in human motion monitoring and human-computer interaction.

The LMFs have applications in energy harvesting. Researcher embedded liquid metal into the hollow fiber-shape silicone rubber and weave these fibers into textiles as triboelectric nanogenerator (LMS) that can harvest mechanical energy from human activities (YANG et al., 2018). The silicone rubber layer serve as the triboelectric and encapsulation material and the liquid metal as the stretchable electrodes. The researcher found that TENG electrical output can be efficiently increased by introducing liquid metal into electrolysis PVD nanofibers as a negative friction layer and thermoplastic polyurethane as a positive friction layer (YU et al., 2018). The peak value of TENG open circuit voltage is up to 1680 V, significantly higher than the current technical value of PVDF-based TENG. The possible reason is that the introduced liquid metal droplets are secondarily polarized inside the fiber, which improves the dielectric constant of the nano-generator and reduces the dielectric loss. However, with the increase in liquid metal content, the mechanical properties of nanofiber membranes decrease gradually. Composite nanofiber membranes containing 2 wt% liquid metal have the best balance of mechanical performance and electrical output balance (SHA et al., 2022). Combining with the coaxial wet spinning process, extremely fine soft triboelectric fibers with polyurethane sheath and liquid metal cores can be produces, and the diameter is only 0.18 mm. In addition, the fiber has good electrical output performance. The output voltage of a 20 cm optical fiber is 20.8 V, which can be used for embroidery or fabric of wearable self-powered sensor (NING et al., 2023).

Liquid metals usually have a low melting point, and we can easily convert liquid metals between solid and liquid states by adjusting the temperature, thus the stiffness of the liquid metals can be greatly changed by converting liquid metals from liquid to solid. Based on the low melting point alloys (47–62°C), medical instruments with variable stiffness can be developed. Researchers injected the liquid metal into a hollow fiber composed of silicone rubber, which were wrapped with helical wires that acted as heaters for melting the metal and greatly change of stiffness of the fiber. In their research, their LMF conformed to the shape of the finger in the soft state and provided support for immobilizing the finger in the stiff state (TONAZZINI et al., 2016). The fibers can be woven into various shapes to be the device for fracture-adaptive splints. Using the property of changing stiffness, liquid metal also has excellent applications in implanting electrodes. The electrode has variable rigidity: High-stiffness electrodes are good for implantation, but are less compatible with human tissue, which may cause tissue damage and signal distortion. Soft electrodes are not suitable for implantation, but they fit well with human tissues, which can reduce damage to the human body and are suitable for long-term monitoring (DENG and LIU, 2014). Because liquid metal can keep certain rigidity under low temperatures while it becomes soft under the environment above the LM melting point. Injecting the liquid metal into a micro-channel to produce LMF, and cooling it to a rigid state can produce electrodes for implanting into the brain. After implantation, the LM is melted to enable the electrodes to conform to the human brain, facilitating long-term monitoring (WEN et al., 2019).

The soft electronics from fibers composed of liquid metal particles and polymers can also respond to external stimulus such as temperature and stretching speed. To obtain a conductor whose resistance is temperature-regulated, liquid metal particles are dispersing in the polymer of bisphenol-A epoxy with a glass transition temperature about 25°C and finally formed into fibers. The temperature-dependent conductors realize several orders of magnitude change in resistance via temperature regulation, and such behavior is fundamentally attributed to the chain dynamics of polymers. Temperature-dependent conductors can work as special thermal conductors, which present programmable and sharp changes in resistance upon temperature fluctuations. The temperature-dependent conductors can serve as thermal conductors to avoid fire, because their resistance will rise sharply when the critical temperature is reached (PENG et al., 2021). In addition to controlling the electrical conductivity of the fibers composed of liquid metal particles and polymers, temperature changes can also change the shape of the fibers. And both the shape and conductivity transition were reversible by heating and cooling (LIU et al., 2020). The liquid metal fibers can also respond to stretching speed. The material maintains electric conductive under low stretching speed, but immediately became an insulator at high stretching speeds. This transformation phenomenon is repeatable, which makes it a promising material for stimulus-response switches (LIU et al., 2021). Injecting the magnetic liquid metal into hollow optical fibers facilitate the use of the fiber in electrical switches for remote magnetic actuation. This fiber with a magnetic liquid metal core has an electrical and magnetic response, which can turn on a circuit and light up an LED through magnetic actuation (HONG et al., 2021).

Liquid metal also can be used to make electroluminescent fiber combined with ZnS micro-particles. Liquid metal-based electroluminescent fibers that can be woven into textiles show potential in healthcare and fashion design. The electroluminescent textile was usually woven by two types of liquid metal fiber: electroluminescent fiber and conductive fiber. Electroluminescent fibers typically contain an elastic polymer core, which is then coated with a liquid metal layer, and a light-emitting layer. Weaving conductive fiber and luminescent fiber forms micrometer-scale electroluminescent units at the contact points. The cross-point between two different fibers form pixels that can be switched on or off independently (MI et al., 2021) (Figure 6F). By doping with different elements, ZnS-based electroluminescent fibers can emit green, blue, or yellow lights. The conductive fiber based on liquid metal can be replaced by a transparent conductor, so that the light emitted by the fiber is not blocked by the conductive fibers (SHI et al., 2021).

## 6 Summary and outlook

In this progress report, we provide an overview of studies to develop breathable liquid metal electronics. We summarized two main strategies to fabricate breathable liquid metal electronics: patterning liquid metal on fiber membranes and weaving liquid metal fibers into breathable e-textiles. Porous fiber membranes can be made by various spinning technologies, such as electrospinning, melt spinning, and air-jet spinning. The appropriate spinning methods should be selected according to the spinning materials, solvent, and environment. An important step in making liquid metal electronics is to pattern the liquid metal on the fiber membrane. The pattern of liquid metals on the porous fiber membranes is achieved by screen printing, inkjet printing, spraying, and dip coating. Before patterning, surface modification is necessary to increase the wettability of fiber membrane by the liquid metal. Preparing liquid metal fibers and weaving them into textiles is also an important strategy to develop liquid metal electronics. Liquid metal fibers are usually prepared by combing liquid metal with different polymers usually spinning methods as summarized above. These fibers usually maintain excellent electrical conductivity and stretchability due to the incorporation of liquid metals. When woven into textiles, liquid metal electronics can be breathable while maintaining stretchability. Those fibers and their textiles have a wide range of applications in soft sensors, nanogenerators, heat dissipation devices, switches, and luminescent wearables.

Although many previous studies have demonstrated the feasibility and superiority of the combination of liquid metal and spinning technology, there are still many challenges that limit the daily application of the breathable liquid metal electronics. For example, the porous structure of the substrate makes it difficult to encapsulate electronic devices, and it will be a huge challenge to make electronic devices waterproof and prevent air oxidation while maintaining air permeability. In addition to encapsulation, it is also important to decrease the diameter of the liquid metal fibers and increase the patterning resolution of liquid metals on porous membrane. Although the spun film is very soft, it is usually non-sticky, and increasing the stickiness of the spun membrane allows electronics to stick to the skin without the need for tape and wristbands. Exciting opportunities remain for developing functional fibers by combining liquid metals with functional polymers, potentially advancing the emerging fields of soft sensors, energy harvesting, and soft robotics.
